# Letter from the Editor in Chief

**DOI:** 10.19102/icrm.2023.14061

**Published:** 2023-06-15

**Authors:** Moussa Mansour


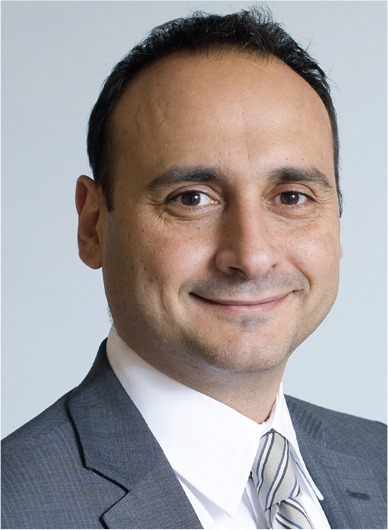


Dear readers,

The annual scientific meeting of the Heart Rhythm Society was held recently in New Orleans, during which many important presentations were given. Atrial fibrillation continues to be an important topic, and various studies on pulsed-field ablation were discussed. Another area of growing interest is left bundle branch area pacing (LBBAP), which has become a popular technique for conduction system pacing. Three studies covering this topic were presented at the late-breaking clinical trial sessions during HRS 2023.

The first and largest study was an observational multicenter investigation completed by Vijayaraman et al.^1^ that enrolled 1,778 patients who met criteria for cardiac resynchronization therapy (CRT). LBBAP was performed in 797 patients, while biventricular pacing was performed in 981. The primary outcome was a composite of time to death or heart failure hospitalization. During a mean follow-up of 33.2 ± 15.5 months, the primary outcome rate was significantly lower in the LBBAP group compared to the biventricular pacing group (20.8% vs. 28%; hazard ratio, 1.495; 95% confidence interval, 1.213–1.842; log-rank *P* < 0.001).

The second study by Diaz et al.^2^ was a multicenter prospective non-randomized trial that enrolled 371 patients. The primary safety outcome was the occurrence of acute and long-term procedure-related complications, and secondary outcomes included improvements in New York Heart Association (NYHA) functional class and electrocardiographic and echocardiographic parameters. LBBAP was performed in 128 patients, while biventricular pacing was performed in 243. At a median follow-up of 340 (interquartile range, 273) days, LBBAP was superior to biventricular pacing.

The third late-breaking clinical trial was a randomized controlled study also completed by Vijayaraman et al.^3^ that enrolled 100 patients randomized to conduction system or biventricular pacing. The primary endpoint was the change in left ventricular ejection fraction at 6 months post-implantation, while secondary endpoints included changes in echocardiographic measurements, NYHA functional class, 6-min walk distance, quality of life, QRS duration, and CRT response. At 6 months of follow-up, conduction system pacing was associated with a greater improvement in left ventricular ejection fraction compared to biventricular pacing (12.4% vs. 8% increase, *P* = 0.02).

These three studies, in addition to other small, previously published ones,^4^ could represent the beginning of a radical shift in CRT. LBBAP appears to be a simpler and less-challenging procedure and, more importantly, is associated with greater improvements in ejection fraction and survival. The results of these studies will need to be confirmed by larger randomized controlled studies, which are underway.

I hope that you enjoy reading this issue of *The Journal of Innovations in Cardiac Rhythm Management*.


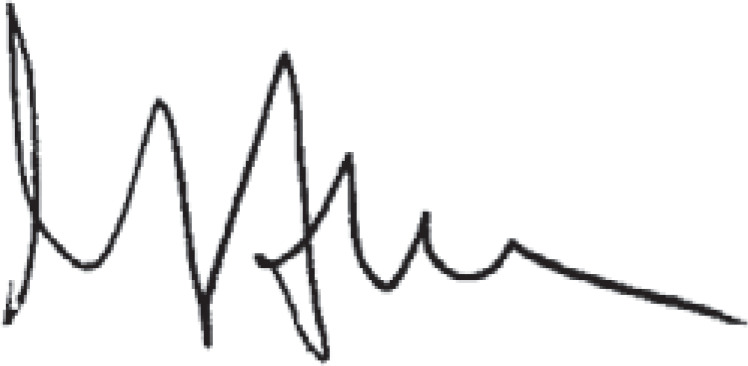


Sincerely,

Moussa Mansour, md, fhrs, facc

Editor in Chief

*The Journal of Innovations in Cardiac Rhythm Management*

MMansour@InnovationsInCRM.com

Director, Atrial Fibrillation Program

Jeremy Ruskin and Dan Starks Endowed Chair in Cardiology

Massachusetts General Hospital

Boston, MA 02114
